# Coenzyme Q10 and Autoimmune Disorders: An Overview

**DOI:** 10.3390/ijms25084576

**Published:** 2024-04-22

**Authors:** David Mantle, Iain P. Hargreaves

**Affiliations:** 1 Pharma Nord (UK) Ltd., Morpeth NE61 2DB, UK; dmantle@pharmanord.co.uk; 2 School of Pharmacy and Biomolecular Sciences, Liverpool John Moores University, Liverpool L3 3AF, UK

**Keywords:** autoimmune disorders, coenzyme Q10, mitochondrial dysfunction, multiple sclerosis, fibromyalgia, antiphospholipid syndrome, Graves’ disease, ulcerative colitis, rheumatoid arthritis

## Abstract

Some 90 autoimmune disorders have been described in medical literature, affecting most of the tissues within the body. Autoimmune disorders may be difficult to treat, and there is a need to develop novel therapeutic strategies for these disorders. Autoimmune disorders are characterised by mitochondrial dysfunction, oxidative stress, and inflammation; there is therefore a rationale for a role for coenzyme Q10 in the management of these disorders, on the basis of its key role in normal mitochondrial function, as an antioxidant, and as an anti-inflammatory agent. In this article, we have therefore reviewed the potential role of CoQ10, in terms of both deficiency and/or supplementation, in a range of autoimmune disorders.

## 1. Introduction

Autoimmune disorders (AIDs) are a category of disease in which the immune system attacks healthy tissues as a result of a dysfunction of the acquired immune system (i.e., aberrant B and T cell activity/autoantibody production), which recognises cellular components as potential antigens [1]. Approximately 90 separate AIDs have been recognised in medical practice, grouped into organ-specific and non-organ-specific types [2]. Disease-specific autoantibodies may be present at an early stage of disease when clinical symptoms are not present in the patient, allowing diagnostic confirmation of the disorder. AIDs vary in prevalence from the very rare to the relatively common, can affect patients of all ages, may predominantly affect women, and can involve most tissue types within the body. Some AIDs may occur primarily in childhood and adolescence (e.g., type 1 diabetes), in middle age (e.g., myasthenia gravis, multiple sclerosis), or in older individuals (e.g., rheumatoid arthritis, primary systemic vasculitis).

In general terms, AIDs are thought to result from a combination of genetic and environmental factors [1]. The genetic predisposition to autoimmunity is not completely understood but may involve multiple genes that regulate the function of immune cell populations, or less frequently, mutations in single genes mediating key regulatory pathways. With regard to environmental factors, infection (particularly viral) is one of the environmental triggers that can result in autoimmunity; other factors include diet, exposure to xenobiotics, and stress. In identical twins, the concordance of AIDs varies between 10 and 70%, illustrating both the role of environmental factors in the pathogenesis of AIDs as well as the potential importance of epigenetic factors.

AIDs have major adverse implications for morbidity and mortality and may be difficult to treat [3]. Current approaches based on immunosuppressive therapies (particularly using corticosteroids) may be associated with a number of adverse effects, and there is scope for the development of alternative therapeutic strategies. In this article, we have therefore reviewed the potential role of coenzyme Q10 (CoQ10) in the pathogenesis and treatment of AIDs (an area of research in which there appears to be a current gap in knowledge) on the basis of its immune function mediating action, its key roles in mitochondrial function, and as an antioxidant. A brief summary of the characteristics of the various AIDs reviewed in this article is given in Table 1.

## 2. Mitochondrial Function in Autoimmune Disorders

A number of studies have provided evidence of ultrastructural changes in mitochondria in autoimmune disorders. For example, a study by Barrera et al. [4] found severe ultrastructural alterations in mitochondria from salivary gland cells in patients with Sjögren’s syndrome. When mitochondria become damaged, leakage of mitochondrial components (particularly mtDNA) into the cell body activates the immune system to produce type 1 interferon, resulting in inflammation and autoimmunity [5]. In addition, a characteristic of dysfunctional mitochondria is the increased production of reactive oxygen free radical species (ROS), one of the consequences of which is T cell activation [6]. Activation of the T lymphocytes results in the production of interferon γ, which in turn can activate macrophages to produce high concentrations of nitric oxide via inducible nitric oxide synthase (iNOS) with concomitant reactive nitrogen species (RNS) generation [7]. ROS and RNS are able to induce mitochondrial respiratory chain (MRC) dysfunction by causing oxidative damage to mitochondrial DNA, mitochondrial membrane phospholipids, and/or the protein subunits of the MRC enzymes [8]. In the autoimmune disease multiple sclerosis (MS), evidence of systemic MRC complex IV deficiency has been reported in the blood mononuclear cells (BMNCs) of patients, which has been associated with increased ROS and RNS generated as a result of the inflammatory response [9]. This loss of MRC function has been associated with the lowered antioxidant status of MS patients, which may render the MRC vulnerable to the detrimental effects of ROS and RNS species [10]. The antioxidant status of patients with other forms of autoimmune disease may also be an important factor to consider, which may contribute to mitochondrial dysfunction and disease progression. Furthermore, a loss of mitochondrial function would impact the lysosome, impairing its ability to maintain its acidic pH and resulting in organelle dysfunction linked to a cellular iron deficiency that is sufficient to trigger inflammatory signalling [11,12].

## 3. CoQ10 and Autoimmune Disorders

Autoimmune disorders reviewed in the present article have been divided into those affecting the cardiovascular, neuromuscular, endocrine, gastrointestinal, skin, respiratory, urinary, and visual systems; this is a somewhat simplified approach in that some disorders may affect more than one system. The potential role of CoQ10 in some 50 autoimmune disorders has been investigated, as listed in Table 2.

### 3.1. Autoimmune Cardiovascular Lymphatic and Blood Clotting Disorders

Autoimmune cardiovascular disorders include rheumatic heart disease, together with the various forms of autoimmune arteritis (giant cell arteritis, polyarteritis nodosa, and Takayasu arteritis), autoimmune vasculitis (Kawasaki disease, Behcets disease, and eosinophilic granulomatosis), antiphospholipid syndrome, and thrombocytopenia [13]. Autoimmune arteritis and vasculitis are disorders in which the immune system attacks particular blood vessels or blood vessel components. Autoimmune rheumatic heart disease is characterised by damage to heart valves caused by an autoimmune inflammatory reaction following a bacterial infection. Autoimmune thrombocytopenia is a disorder characterised by reduced levels of blood platelets. Castleman disease is a disorder characterised by the overgrowth of cells within lymph nodes [14]. Of the above disorders, only antiphospholipid syndrome has been investigated with regard to CoQ10 deficiency/supplementation, as described in the following section.

**Antiphospholipid syndrome** (APS) is a systemic autoimmune disease that is characterised by pregnancy morbidity as well as, in some cases, a hypercoagulable state of the venous or arterial vasculature associated with the persistence of antiphospholipid antibodies [15]. Mitochondrial dysfunction, oxidative stress, and inflammation have been implicated in the pathogenesis of APS [16,17,18]. As an alternative to anticoagulant-based therapy, research has focused on anti-inflammatory agents such as CoQ10. In a randomised controlled trial, patients were supplemented with the reduced form of CoQ10 (ubiquinol; 200 mg/day for 1 month); endothelial function was improved, and expression of pro-thrombotic and pro-inflammatory mediators was reduced [19]. Because of the absence of significant adverse effects, it is suggested that CoQ10 has potential as an adjunct to standard therapy in the treatment of APS.

### 3.2. Autoimmune Neuromuscular and Musculoskeletal Disorders

Autoimmune disorders of the CNS/skeletal muscles include fibromyalgia [20], Guillain–Barre syndrome [21], Lambert–Eaton syndrome [22], multiple sclerosis [23], myasthenia gravis [24], neuromyelitis optica [25], polymyositis [26], restless legs syndrome [27], stiff person syndrome [28], and Sydenham’s chorea [29]. Many of these disorders involve some form of mitochondrial dysfunction, oxidative stress, and/or inflammation [30,31,32]. There is therefore a potential role for supplementation with CoQ10 in the treatment of these disorders, given its key roles in normal mitochondrial function as an antioxidant and an anti-inflammatory agent. However, with the exception of fibromyalgia and multiple sclerosis, the role of CoQ10 deficiency or supplementation in the pathogenesis and treatment of these disorders has, to date, not been investigated. The role of CoQ10 deficiency and supplementation in the treatment of fibromyalgia and multiple sclerosis has been described in the following sections.

**Fibromyalgia** is a disorder characterised by both fatigue and muscle pain. Fibromyalgia patients have been shown to have depleted tissue levels (typically 40–50% of normal) of CoQ10, together with increased levels of mitochondrial dysfunction, oxidative stress, and inflammation, both in adults [33] and juveniles [34]. A randomised controlled clinical study by Cordero et al. [33] in 20 fibromyalgia patients found supplementation with CoQ10 (300 mg/day for 40 days) significantly reduced (by more than 50%) pain and fatigue; there was a corresponding improvement in mitochondrial ATP energy generation and reduced oxidative stress and inflammation. In this study, psychopathological symptoms (including depression) were also significantly improved; this was linked to the effect of supplemental CoQ10 in reducing oxidative stress and inflammation, as well as increased levels of serotonin [35,36]. In addition, Cordero et al. [37] correlated headache symptoms with reduced CoQ10 levels and increased oxidative stress in fibromyalgia patients, with headache symptoms and oxidative stress levels significantly improving following CoQ10 supplementation (300 mg/day for 3 months). Finally, in juvenile fibromyalgia patients, Miyamae et al. [34] reported that CoQ10 supplementation (100 mg/day for 3 months) significantly improved fatigue.

**Multiple sclerosis** (MS) is an auto-immune disorder in which the immune system attacks the protective myelin protein sheath surrounding the axons of neurons [38]. The resulting inflammation damages and scars the sheath, causing problems with nerve transmission. The conventional treatment of MS involves the use of drugs that modulate immune function. In the relapsing-remitting disease phase, immunomodulating drugs such as beta-interferon can be used to reduce the frequency of relapses [39], and symptom flare-ups can be controlled using anti-inflammatory drugs such as corticosteroids [40]. However, such drugs may have a number of severe side effects, which limits their use. In addition, once patients enter the progressive phase of disease, currently available drugs may provide relatively little clinical benefit. There is therefore a need to develop alternative therapeutic strategies for this disorder. Since there is evidence for the involvement of mitochondrial dysfunction, oxidative stress, and inflammation in the pathogenesis of this disorder, supplementation with CoQ10 has novel therapeutic potential.

With regard to CoQ10 deficiency in MS, Gironi et al. [41] reported reduced blood levels of CoQ10 (mean value 0.48 μg/mL versus 0.62 μg/mL for normal controls) in a series of 87 patients with MS. Similarly, Steen et al. [42] found serum CoQ10 levels to be significantly reduced (mean value of 0.54 μg/mL versus 0.84 μg/mL for normal controls) in a set of 20 MS patients. In contrast to the above studies, de Bustos et al. [43] found no significant difference in serum CoQ10 levels between MS patients and normal controls.

Both pre-clinical and clinical studies have provided evidence that CoQ10 supplementation may be of therapeutic benefit in MS. In a well-established animal model for studying demyelination-remyelination (MS induced by cuprizone in mice), administration of CoQ10 (150 mg/kg/day for 12 weeks) significantly reduced the levels of oxidative stress and inflammatory biomarkers (total antioxidant capacity, interleukin-6, tumour necrosis factor-alpha) in corpus callosum while enhancing remyelination [44]. In a different animal model system of MS (autoimmune encephalomyelitis model in mice), administration of CoQ10 (10 mg/kg/day for 3 weeks) significantly reduced brain (corpus callosum) levels of the inflammatory marker tumour necrosis factor-alpha while significantly improving clinical symptoms [45].

Several clinical studies supplementing CoQ10 in MS patients have demonstrated significant benefits in reducing oxidative stress and inflammation. In a randomised controlled trial comprising 48 patients with relapsing-remitting multiple sclerosis, supplementation with CoQ10 (500 mg/day for 3 months) resulted in a significant reduction in blood levels of the inflammatory biomarkers interleukin-6 (IL-6) and tumour necrosis factor-alpha (TNF-alpha). CoQ10 supplementation also improved symptoms of fatigue (assessed via the fatigue severity scale) and depression (assessed via the Beck depression inventory) [46,47].

In an open-label study of 60 patients with relapsing-remitting multiple sclerosis by Moccia et al. [48], supplementation with CoQ10 (100 mg/day for 3 months) reduced serum 8-hydroxy-2-deoxyguanosine and protein carbonyl levels as biomarkers of oxidative stress. CoQ10 supplementation also reduced blood levels of proinflammatory cytokines, including interleukins (IL1-alpha, IL-2R, IL-9, and IL-17F) and tumour necrosis factor-alpha. In addition, CoQ10 supplementation was associated with a lower Expanded Disability Status Scale, fatigue severity scale, Beck’s depression inventory, and the visual analogue scale for pain.

**Rheumatoid arthritis (RA)** is a chronic inflammatory disorder primarily affecting joints, resulting from the development of autoantibodies against components of the joint lining [49]. To date, there have been two randomised controlled trials supplementing CoQ10 in RA. In a study comprising 44 patients with RA, supplementation with CoQ10 (100 mg/day for 3 months) reduced the levels of oxidative stress and inflammatory markers (malondialdehyde and tumour necrosis factor) [50]. In a study comprising 54 patients with RA, supplementation with CoQ10 (100 mg/day for 2 months) attenuated disease severity, associated with a reduction in serum matrix metalloproteinase activity [51]. Several studies have described the beneficial effects of supplementary CoQ10, alone or in combination, in animal models of RA. For example, in zymosan-induced arthritis in mice, administration of CoQ10 reduced inflammation and disease severity [52]. A combination of CoQ10 and metformin improved mitochondrial function and reduced disease severity in collagen-induced arthritis in mice [53]. Similarly, in a rat model of adjuvant-induced arthritis, a combination of CoQ10 and omega-3 polyunsaturated fatty acids improved mitochondrial function and reduced inflammation [54].

### 3.3. Autoimmune Endocrine Disorders

Autoimmune disorders of the endocrine system [55] include Addison’s disease (adrenal insufficiency), autoimmune oophoritis (ovarian insufficiency), autoimmune orchitis (inflammation of testes and anti-sperm antibodies), type I diabetes (insulin insufficiency), Graves’ disease (hyperthyroidism), Hashimoto’s disease (hypothyroidism), and Sjogren’s disease (affects fluid secreting glands). Of the above disorders, only Graves’ disease and type I diabetes have been investigated with regard to CoQ10 deficiency or supplementation, as summarised in the following sections.

Type 1 diabetes is an autoimmune disorder in which insulin-secreting beta cells in the pancreas are destroyed by the immune system. Approximately 10% of diabetes cases are type I, which tends to occur earlier in life. Mitochondrial dysfunction, oxidative stress, and inflammation have been implicated in the pathogenesis of type I diabetes, providing a rationale for the potential role of supplemental CoQ10 in mediating this disorder. The situation with regard to possible CoQ10 deficiency is unclear, since blood CoQ10 levels have variously been reported to be increased [56] and decreased [57,58] in patients with type I diabetes.

Relatively little work has been published regarding the effect of CoQ10 supplementation on type I diabetes. In a randomised controlled trial comprising 34 patients with type I diabetes, supplementation with CoQ10 (100 mg/day for 3 months) had no significant benefit on glycaemic control (blood glucose level, HbA1c, insulin dose) [59]. Similarly, Serag et al. [60] reported supplementation with 100 mg/day CoQ10 had no significant effect on endothelial dysfunction (soluble intracellular adhesion molecule-1) or glycaemic control (blood glucose and HbA1c) in an open-label study comprising 49 type I diabetes paediatric patients. However, supplementation with CoQ10 (200 mg/day for 3 months) in a group of 23 patients with type 1 diabetes significantly reduced circulatory levels of the inflammatory marker human beta-defensin 1 while improving natural killer cell activity [61].

**Thyroid disorders.** There are two autoimmune disorders of the thyroid, i.e., Graves’ disease and Hashimoto’s disease. Graves’ disease is a form of hyperthyroidism characterised by overproduction of thyroid hormones, resulting from autoimmune damage to the thyroid; autoantibodies directed against the thyrotropin receptor bind to and activate the receptor, causing the autonomous production of thyroid hormones [62].

Hashimoto’s disease is a form of hypothyroidism resulting from autoimmune damage to the thyroid, in which autoantibodies are directed against the thyroid antigens thyroid peroxidase and thyroglobulin [63]. It is worth noting that thyroid hormones regulate cellular energy metabolism via their effects on mitochondrial function [64,65]. Mitochondria are major sites of triiodothyronine accumulation within cells, where it exerts a direct effect on mitochondrial activity and energy metabolism [66]. Mitochondria are a major source of free radical production within cells, and the accelerating effect of triiodothyronine on basal metabolism results in an increased production of free radicals. The hypermetabolic state present in hyperthyroidism results in excessive free-radical-induced oxidative stress within cells; in contrast, the hypometabolic state induced by hypothyroidism leads to a decrease in free radical production [67,68,69].

A number of studies have described significantly reduced levels of CoQ10 in blood [70,71,72,73,74,75,76,77] or in thyroid tissue [78]. In patients with hyperthyroidism, mean serum CoQ10 levels (16 patients) of 0.37 ± 0.17 µg/mL (versus 1.01± in normal controls) were reported by Ogura et al. [70], and in a set of 20 patients as 0.28 ± 0.03 µg/mL (versus 0.65 ± 0.08 µg/mL in normal controls) by Suzuki et al. [71]. In the latter study, 12 of the hyperthyroid patients were supplemented with CoQ10 (120 mg/day for 1 week); the mean serum CoQ10 level was elevated to 0.66 ± 0.05 µg/mL, with a corresponding improvement in some parameters of cardiac function, namely stroke volume and systolic time intervals (pre-ejection time and left ventricular ejection time/pre-ejection time ratio).

In the study by Mancini et al. [72], a mean plasma CoQ10 value of 0.49 ± 0.03 µg/mL was reported in a set of eight hyperthyroid patients (versus a normal plasma range of 0.70–1.00 µg/mL). Similarly, in hyperthyroid patients, reduced mean plasma CoQ10 levels were reported by Grossi et al. [73] (21 patients; 0.27 ± 0.13 µg/mL vs. 0.80 ± 0.20 µg/mL for normal controls) and Pandolfi et al. [74] (0.51 ± 0.35 µg/mL versus 0.73 ± 0.16 µg/mL for normal controls). Bianchi et al. [75] reported a mean plasma CoQ10 level of 0.63 µmol/L (versus 0.89 µmol/L for normal controls) in a series of 22 hyperthyroid patients; the decrease in plasma CoQ10 levels correlated with increased oxidative stress, quantified via measurement of plasma lipid peroxide levels. In a study by Jiang et al. [76], the mean plasma CoQ10 level (0.46 µg/mL) in a series of 38 hyperthyroid patients was significantly reduced compared to the corresponding value in normal controls (0.65 µg/mL). A mean plasma CoQ10 level of 0.46 µmol/L (versus 0.74 µmol/L for normal controls) was reported by Menke et al. [77] in 12 children with hyperthyroidism; the plasma CoQ10 level was still significantly reduced in hyperthyroid subjects when expressed relative to cholesterol (0.169 µmol/mol) compared to controls (0.210 µmol/mol), demonstrating a lipid-independent phenomenon (lipoprotein degradation may be increased in hyperthyroidism [79]. In thyroid tissue obtained by surgical resection from eight patients with Graves’ disease, the level of CoQ10 (12.3 ± 3.4 mg/gww) was significantly reduced compared to the CoQ10 level in normal thyroid tissue (17.0 ± 3.4 mg/gww) [78].

Possible causes of the low tissue levels of CoQ10 in hyperthyroid patients include the following: (i) decreased synthesis resulting from competition for tyrosine, which is utilised both in CoQ10 and thyroxine biosynthesis; (ii) reduced levels of CoQ10 resulting from increased oxidative stress associated with hyperthyroidism; (iii) decreased levels of lipoprotein CoQ10 carriers in blood, either from increased degradation or reduced release from the liver. CoQ10 deficiency has been suggested as a factor in complications of hyperthyroidism, including heart failure. Pre-clinical research and a small clinical study indicate CoQ10 supplementation may help improve cardiac performance in those with hyperthyroidism [68,71,80]. A therapeutic regime supplementing CoQ10 (60 mg/day ongoing) for the correction of CoQ10 deficiency in patients with thyroid disease has been described by Moncayo and Moncayo [81].

In contrast to the situation in hyperthyroidism, patients with hypothyroidism may have similar circulatory levels of CoQ10 to normal subjects [70] or substantially increased levels [71,76], precluding the necessity of CoQ10 supplementation.

### 3.4. Gastrointestinal Autoimmune Disorders

Autoimmune disorders of the gastrointestinal system [82] include autoimmune enteropathy (chronic diarrhoea resulting from small intestinal villous atrophy)*,* autoimmune hepatitis (inflammation of the liver), celiac disease (inflammation of the small intestine in response to eating gluten protein), Crohn’s disease (inflammation/ulceration of the digestive tract), pernicious anaemia (vitamin B12 deficiency), and ulcerative colitis (inflammation/ulceration of the colon and bowel). Of the above disorders, only ulcerative colitis has been investigated with regard to CoQ10 deficiency or supplementation. In a randomised controlled clinical trial, supplementation with CoQ10 (200 mg/day for 8 weeks) significantly reduced levels of inflammatory markers and disease severity and improved quality of life in patients with mild to moderate ulcerative colitis [83]. Several studies in animal models of ulcerative colitis have indicated a beneficial role for CoQ10 supplementation. Administration of CoQ10 (10 mg/kg for 8 days prior to induction) was found to protect against acetic acid-induced colitis in rats via its antioxidant, anti-inflammatory, and energy restoration actions [84]. In a dextran sodium sulphate-induced mouse model of ulcerative colitis, co-administration of the CoQ10 analogue idebenone (200 mg/kg) significantly prevented body weight loss and improved the disease activity index, colon length, and histopathological score [85]. In a rat model of dextran sodium sulphate-induced ulcerative colitis, pre-administration of a combination of CoQ10 and Aloe vera improved inflammation, electrical/mechanical impairment in the gut, and a number of oxidative stress parameters [86]. Protection against ulcerative colitis in a rat model induced by iodoacetamide via the antioxidant and anti-inflammatory action of CoQ10 was reported by Ewees et al. [87].

### 3.5. Autoimmune Disorders of the Skin

Autoimmune disorders of skin [88] include alopecia areata (immune attack on hair follicles), autoimmune angioedema (swelling of face resulting from antibodies against complement related proteins), Parry–Romberg syndrome (characterised by the progressive degeneration of tissues of one side of the face), autoimmune urticaria (raised skin rash resulting from immune attack on mast cells), autoimmune pemphigoid (blistered skin resulting from immunoglobulin antibodies), dermatomyositis (inflammation of skin and muscles resulting from antibodies against histidyl tRNA synthase), lichen planus (mucocutaneous inflammation resulting from anti-keratinocyte antibodies), lupus (systemic inflammation linked to anti-nuclear antibodies), psoriasis (skin scaling resulting from immune attack on skin cells), scleroderma (inflammation and thickening of skin linked to anti-nuclear antibodies) and vitiligo (skin discolouration resulting from antibodies against melanocytes). With regard to clinical studies, there has been one study relating to CoQ10 and lichen planus and two studies on psoriasis.

A randomised controlled trial comprising 34 patients with lichen planus found topical application of a mucoadhesive CoQ10 formulation three times daily for one month significantly reduced pain and lesion size [89].

In a prospective double-blind controlled study comprising 24 patients with psoriasis, supplementation with CoQ10 (100 mg/day for 3 months) resulted in improvements in the psoriasis area, severity index, and dermatology life quality index, respectively, compared to placebo [90]. In a controlled study comprising 58 patients with psoriasis, treatment with CoQ10 (50 mg/day for 30 days) in combination with vitamin E and selenium resulted in reduced levels of oxidative stress markers and improved clinical status compared to placebo [91].

In vitiligo, Passi et al. [92] reported reduced levels of CoQ10 (in ubiquinol form), together with other antioxidants, in epidermal samples from 15 vitiligo patients, compared to normal controls.

Lupus is an autoimmune disorder that can affect various tissues, including the skin, joints, kidneys, and other organs [93]. Lupus is associated with mitochondrial dysfunction, oxidative stress, and inflammation [94,95,96] and therefore may be amenable to CoQ10 therapy. To date, there have been no randomised controlled trials of supplemental CoQ10 in lupus; however, the CoQ10 analogues idebenone and MitoQ improved clinical and immunological characteristics in mouse models of lupus, suggesting a potential role for CoQ10 in the treatment of lupus patients. In the lupus-susceptible NZM2328 mouse model, oral supplementation with idebenone (1 g/kg for 8 weeks) decreased systemic inflammation, improved renal function, and reduced mortality [97]. In lupus-prone MRL-1 pr mice, the addition of MitoQ (200 μM) to drinking water for 11 weeks reduced oxidative stress levels and renal tissue damage [98].

### 3.6. Autoimmune Respiratory Disorders

Goodpasture’s syndrome is a disorder characterised by inflammation of the glomeruli filtering structures of the lungs and kidneys, associated with the production of anti-glomerular basement membrane antibodies [99]. Sarcoidosis is an autoimmune disorder characterised by the development of granulomas in lung tissue [100]. Neither of these disorders has been investigated with regard to the potential deficiency or supplementation with CoQ10.

### 3.7. Autoimmune Urinary Disorders

IgA nephropathy (also known as Berger’s disease) is a renal disorder resulting from excessive deposition of IgA protein within the glomeruli [101]. Metabolic profiling found the biosynthesis of CoQ10 to be altered in a set of 65 patients with IgA nephropathy, compared to controls [102]. Administration of antroquinolol, a naturally occurring ubiquinone derivative, was reported to inhibit the development of renal lesions in mice with IgA nephropathy [103].

### 3.8. Autoimmune Visual Disorders

Autoimmune retinopathy is a disorder resulting from the loss of photoreceptor function following the development of antibodies against retinal proteins [104]. Autoimmune uveitis involves inflammation of the inner eye (between the sclera and the retina) resulting from the development of antibodies against several ocular antigens [105]. Cogan’s syndrome is an autoimmune disorder affecting vision and hearing, resulting from the development of antibodies against corneal and inner ear antigens [106]. In Susac’s syndrome, the immune system attacks endothelia lining small blood vessels supplying blood to the brain, retina, and inner ear [107]. Tolosa–Hunt syndrome is characterised by painful headaches and difficulty moving the eyes, linked to the development of antibodies against the water channel protein aquaporin 4 [108]. None of the above disorders have been investigated to date in terms of possible CoQ10 deficiency or supplementation.

## 4. Conclusions

**Figure 1:**
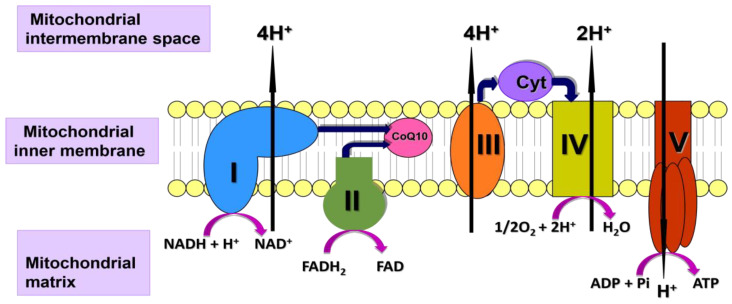
Diagram of the mitochondrial electron transport chain (ETC) showing the involvement of CoQ10 in the process of oxidative phosphorylation. ETC Complexes I–IV (I–IV) and the electron carriers coenzyme Q10 (CoQ10), cytochrome c (Cyt), and protons (H^+^).

In general terms, autoimmune disorders are characterised by mitochondrial dysfunction, oxidative stress, and inflammation [6,109]. In principle, administration of supplementary CoQ10 could be of clinical benefit in such disorders, given its key roles in normal mitochondrial function, as an antioxidant, and as an anti-inflammatory agent. CoQ10 has a key role as an electron carrier (from complex I and II to complex III) in the mitochondrial electron transport chain during oxidative phosphorylation (Figure 1). CoQ10 also serves as an important lipid-soluble antioxidant, protecting mitochondria from oxidative damage induced by reactive oxygen-free radical species generated during the oxidative phosphorylation process. Of particular note, CoQ10 performs a number of cellular functions relevant to the functioning of the immune system. Through its role in oxidative phosphorylation within the mitochondria, CoQ10 contributes to the supply of cellular energy. The immune system has considerable energy requirements. Therefore, an adequate supply of CoQ10 is needed to enable the various cell types of the immune system to function optimally. CoQ10 also functions as an important lipid-soluble antioxidant that protects cellular membranes and circulatory lipoproteins from free radical-induced oxidative damage. Moreover, the antioxidant action of CoQ10 may protect phagocytic cells from possible self-destruction caused by their own generation of free radicals. CoQ10 modulates directly the action of genes involved in inflammation. It may have a role in controlling the release of pro-inflammatory cytokines in disorders in which cytokine release is required (Mantle et al., 2021 [110]). With regard to efficacy, for some autoimmune disorders, for example, fibromyalgia and multiple sclerosis, deficiency of CoQ10 has been identified, and the beneficial effects of CoQ10 supplementation have been described. 

The link between mitochondrial dysfunction and the autoimmune disorders reviewed in this article has been summarised in Table 3, together with the outcome of CoQ10 supplementation in the respective disorders. However, it is apparent from Table 2 that the role of CoQ10 (in terms of deficiency or supplementation) has yet to be investigated for many of the recognised forms of autoimmune disease, and this remains a major area for future research. With regards to safety, none of the clinical studies identified in this article reported any significant adverse effects following CoQ10 supplementation. In more general terms, the safety of supplemental CoQ10 (in both animal models and humans) has been assessed by Hidaka et al. (2008) [111]. Results from animal-based studies found supplemental CoQ10 to have low toxicity, with no evidence of effects on development or mutagenicity and no evidence of the induction of serious adverse effects in humans. In addition, more than 200 randomised controlled clinical trials are currently listed on Medline in which supplementary CoQ10 has been administered in a variety of disorders in various dosages (up to 3000 mg/day) and for various time periods (up to 5 years); in none of these studies were any serious adverse effects attributable to CoQ10 reported. There is no published evidence that supplementary CoQ10 interferes in an adverse way with medicines likely to be prescribed for patients with autoimmune disorders; however, supplementation with CoQ10 may facilitate the use of reduced steroid dosages in other types of disorders, for example, asthma [112]. In general terms, there are very few reports of supplementary CoQ10 interfering with prescribed medicines, being limited to older anti-coagulants or antihypertensive drugs.

## Figures and Tables

**Table 1:** Summary of characteristics of autoimmune disorders reviewed in this article.

System/Disorder	Target Tissue	Autoantibody Targets	Prevalence
**Cardiovascular/lymphatic** Autoimmune arteritis	Arteries	Endothelial and cytoskeletal proteins	10–100/100,000
Autoimmune vasculitis	Blood vessels	ANCA (neutrophil cytoplasm)	20–200/100,000
Autoimmune rheumatic heart disease	Heart valves	Rheumatoid factor and citrullinated proteins	2/1000
Antiphospholipid syndrome	Blood	Cardiolipin and beta-2 glycoprotein	50/100,000
Thrombocytopenia	Blood platelets	Platelet membrane glycoproteins	9/100,000
Castleman disease	Lymph nodes	Nucleus, double-stranded DNA, and voltage-gated potassium channels	1/100,000
**Neuromuscular:** Fibromyalgia	Muscles	Satellite glia cells	2–4/100
Guillain–Barre syndrome	Nerves	Gangliosides	5–10/100,000
Lambert–Eaton syndrome	CNS (neuromuscular junction)	Voltage-gated calcium channels	1–2/1,000,000
Multiple sclerosis	CNS	Myelin	30–90/100,000
Myasthenia gravis	CNS (neuromuscular junction)	Nicotinic acetylcholine post-synaptic receptors	15–20/100,000
Neuromyelitis optica	Optic nerves	Aquaporin-4	1–5/100,000
Restless legs syndrome	CNS	Interferon α	5–10/100
Stiff-person syndrome	CNS	Glutamic acid decarboxylase	1–2/1,000,000
Sydenham’s chorea	Brain	Basal ganglia	Rare
**Musculoskeletal:** Polymyositis	Muscles	Jo-1	10/100,000
Rheumatoid arthritis	Joints	Rheumatoid factor, cyclic citrullinated peptide-2, and carbamylated protein	0.5–1/100
**Endocrine:** Addison’s disease	Adrenals	21-hydroxylase	1/10,000
Autoimmune oophoritis	Ovaries	Multiple ovarian antigens	4% of females with primary ovarian insufficiency
Autoimmune orchitis	Testes	Anti-sperm antibodies	Not accurately known, but very rare
Diabetes type I	Pancreas	Islet cells, insulin, glutamic acid decarboxylase, and tyrosine phosphatase	1–4/1000
Graves’ disease	Thyroid	TSH receptor, thyroglobulin, and thyroid peroxidase	1/100
Hashimoto’s disease	Thyroid	Thyroglobulin and thyroid peroxidase	1/100
Sjogren’s disease	Salivary/lacrimal glands	SS-A and SS-B	0.1–4/100
**Gastrointestinal:** Autoimmune enteropathy	Small intestine	Enterocytes	<1/100,000
Autoimmune hepatitis	Liver	Various, including nuclear, smooth muscle	1/10,000
Celiac disease	Small intestine	Transglutaminase and endomysium	1/100
Crohn’s disease	Digestive tract	Neutrophils and saccharomyces cerevisiae	2/1000
Pernicious anaemia	Stomach	Parietal cells and intrinsic factor	1/1000
Ulcerative colitis	Colon/rectum	Integrin alpha-v/beta-6	2–300/100,000
**Skin:** Alopecia areata	Hair follicles	Hair follicle antigens	1/1000
Autoimmune angioedema	Face	C1-1NH	0.2–1/100,000
Parry–Romberg syndrome	Face	Nucleus and rheumatoid factor	0.3–1/250,000
Autoimmune urticaria	Skin	IgE and high-affinity IgE receptor	1/1000
Autoimmune pemphigoid	Skin	Structural proteins of the epidermal–dermal junction	1.5/10,000
Dermatomyositis	Skin and muscles	Mi-2 nuclear antigen and Jo-1	0.2–1/10,000
Lichen planus	Skin	Desmoglein	1/100
Lupus	Skin	Nucleus and double-stranded DNA	20–100/100,000
Psoriasis	Skin	LL-37, ADAMTS-L5	1–2/100
Scleroderma (localised)	Skin	Centromeres, topoisomerase	10–30/100,000
Vitiligo	Skin	Melanocytes	0.5–2/100
**Respiratory:** Goodpasture’s syndrome	Lungs and kidneys	Alveolar or glomerular basement membranes	1/100,000
Sarcoidosis (pulmonary)	Lungs	Elevated IgG and IgM autoantibodies	10–40/100,000
**Urinary:** IgA nephropathy	Kidneys	IgA1 (glomerulus)	3/100,000
**Visual:** Autoimmune retinopathy	Retina	Retinal proteins	Extremely rare
Autoimmune uveitis	Uvea	Neurofilament-M	20–60/100,000
Cogan’s syndrome	Eye and inner ear	Sensory epithelia and endothelia	Not accurately known, rare
Susac’s syndrome	Retina and inner ear	Endothelia	Not accurately known, rare
Tolosa–Hunt syndrome	Orbit	Aquaporin-4	Prevalence unknown; incidence estimated at 1–2/1,000,000

**Table 2:** Status of CoQ10 deficiency or supplementation studies in autoimmune disorders.

System	Disorder	CoQ10 Deficiency	CoQ10 Supplementation
Cardiovascular	Autoimmune arteritis	Not investigated	Not investigated
	Autoimmune vasculitis	Not investigated	Not investigated
	Autoimmune rheumatic heart disease	Not investigated	Not investigated
	Antiphospholipid syndrome	Not investigated	See Section 3.1
	Thrombocytopenia	Not investigated	Not investigated
	Castleman disease	Not investigated	Not investigated
Neuromuscular	Fibromyalgia	See Section 3.2	See Section 3.2
	Guillain–Barre syndrome	Not investigated	Not investigated
	Lambert–Eaton syndrome	Not investigated	Not investigated
	Multiple sclerosis	See Section 3.2	See Section 3.2
	Myasthenia gravis	Not investigated	Not investigated
	Neuromyelitis optica	Not investigated	Not investigated
	Restless legs syndrome	Not investigated	Not investigated
	Stiff-person syndrome	Not investigated	Not investigated
	Sydenham’s chorea	Not investigated	Not investigated
Musculoskeletal	Polymyositis	Not investigated	Not investigated
	Rheumatoid arthritis	See Section 3.2	See Section 3.2
Endocrine	Addison’s disease	Not investigated	Not investigated
	Autoimmune oophoritis	Not investigated	Not investigated
	Autoimmune orchitis	Not investigated	Not investigated
	Diabetes type I	See Section 3.3	See Section 3.3
	Graves’ disease	See Section 3.3	See Section 3.3
	Hashimoto’s disease	Not investigated	Not investigated
	Sjogren’s disease	Not investigated	Not investigated
Gastrointestinal	Autoimmune enteropathy	Not investigated	Not investigated
	Autoimmune hepatitis	Not investigated	Not investigated
	Celiac disease	Not investigated	Not investigated
	Crohn’s disease		
	Pernicious anaemia	Not investigated	Not investigated
	Ulcerative colitis	See Section 3.4	See Section 3.4
Skin	Alopecia areata	Not investigated	Not investigated
	Autoimmune angioedema	Not investigated	Not investigated
	Parry–Romberg syndrome	Not investigated	Not investigated
	Autoimmune urticaria	Not investigated	Not investigated
	Autoimmune pemphigoid	Not investigated	Not investigated
	Dermatomyositis	Not investigated	Not investigated
	Lichen planus	Not investigated	See Section 3.5
	Lupus	Not investigated	See Section 3.5
	Psoriasis	Not investigated	See Section 3.5
	Scleroderma (localised)	Not investigated	Not investigated
	Vitiligo	See Section 3.5	Not investigated
Respiratory	Goodpasture’s syndrome	Not investigated	Not investigated
	Sarcoidosis	Not investigated	Not investigated
Urinary	IgA nephropathy		
Visual	Autoimmune retinopathy	Not investigated	Not investigated
	Autoimmune uveitis	Not investigated	Not investigated
	Cogan’s syndrome	Not investigated	Not investigated
	Susac’s syndrome	Not investigated	Not investigated
	Tolosa–Hunt syndrome	Not investigated	Not investigated

**Table 3:** Mitochondrial dysfunction and autoimmune disease outcomes.

Reference	Disorder	Mitochondrial Disorder
Rai (2021) [4]	Autoimmune diseases in general	Leakage of damaged mitochondrial components (particularly mtDNA) into the cells activates the production of type 1 interferon and results in inflammation and autoimmunity
Chavez and Tse (2021) [5]	Autoimmune disorders in general	Increased production of reactive oxygen species results in T cell activation
Hargreaves et al. (2018) [9]	Multiple sclerosis	Deficiency of Complex IV and increased oxidative stress
Barrera et al. (2021) [4]	Sjögren’s syndrome	Ultrastructural alterations observed in mitochondria from salivary gland cells
Shah et al. (2014), Aringer et al. (2019), and Yang et al. (2020) [94,95,96]	Lupus	Pathogenesis associated with mitochondrial dysfunction, oxidative stress, and systemic inflammation
Perez-Sanchez et al. (2017) [19]	Antiphospholipid syndrome	CoQ10 reduced levels of inflammatory markers, and endothelial function improved
Farsi et al. (2021) [83]	Ulcerative colitis	CoQ10 reduced levels of inflammatory markers and disease severity
Cordero et al. (2013) [33]	Fibromyalgia	CoQ10 increased mitochondrial ATP energy generation and reduced oxidative stress and inflammation
Sanoobar et al. (2015) [46]	Multiple sclerosis	CoQ10 reduced blood levels of the inflammatory biomarkers IL-6 and TNF-alpha
Abdollahzad et al. (2015) [50]	Rheumatoid arthritis	CoQ10 reduced blood levels of oxidative stress and inflammatory markers
Naito et al. (1986) [86]	Graves’ disease	CoQ10 improved cardiac performance
Kharaeva et al. (2009) [91]	Psoriasis	CoQ10, together with vitamin E and selenium, reduces levels of oxidative stress
Abdelsamie et al. (2023) [89]	Lichen planus	Topical application of CoQ10 reduced pain

## Data Availability

Not applicable.
